# RNA Capping, Replication Complex Assembly, and Translational Regulation in Chikungunya Virus: Molecular Mechanisms and Antiviral Opportunities

**DOI:** 10.7759/cureus.113622

**Published:** 2026-07-29

**Authors:** Rohit R Patil, Satyajeet Pawar, Satish Patil, Dilip D Hinge

**Affiliations:** 1 Department of Molecular Biology and Genetics, Krishna Institute of Medical Sciences, Krishna Vishwa Vidyapeeth (Deemed to be University), Karad, IND; 2 Department of Microbiology, Krishna Institute of Medical Sciences, Krishna Vishwa Vidyapeeth (Deemed to be University), Karad, IND

**Keywords:** alphavirus, antiviral targets, chikungunya virus, host shutoff, nsp1, replication complex, rna capping, translational regulation

## Abstract

Chikungunya virus (CHIKV) is an emerging mosquito-borne alphavirus that has become a significant global public health concern due to its rapid spread and the debilitating acute and chronic joint pain (arthralgia) it causes. Understanding the molecular events that regulate CHIKV RNA synthesis and gene expression is essential for identifying vulnerable stages in the viral life cycle and guiding the development of targeted therapies. This narrative review summarises key aspects of CHIKV molecular biology, focusing on RNA capping, genome replication, and translation. CHIKV RNA capping is primarily facilitated by non-structural protein 1 (nsP1), which has both methyltransferase and guanylyl transferase activities. These activities are necessary for producing the 5' type-0 cap structure (m⁷GpppN), which stabilises viral RNA, enhances translation efficiency, and aids in immune evasion. Viral genome replication takes place within membrane-derived replication spherules that compartmentalise replication complexes composed of nsP1-nsP4 and host cofactors, protecting RNA intermediates from detection by the innate immune system. The RNA-dependent RNA polymerase nsP4 drives the synthesis of negative-strand templates, followed by the amplification of positive-sense genomic RNA and the transcription of 26S sub-genomic RNA for structural protein production. CHIKV also modifies host translation and antiviral signalling to maintain efficient viral protein synthesis. These findings demonstrate that nsP1, nsP2, and nsP4 are promising antiviral targets. They emphasise the importance of understanding CHIKV's molecular mechanisms to develop effective therapeutic strategies against CHIKV and related alphaviruses.

## Introduction and background

Chikungunya virus (CHIKV) is an arthropod-borne virus (arbovirus) that belongs to the genus Alphavirus within the family Togaviridae. First isolated during an outbreak in Tanzania in 1952, CHIKV has become a significant global public health concern due to its rapid geographical spread and its capacity to cause large-scale epidemics. The virus is primarily transmitted through the bites of infected mosquitoes, specifically *Aedes aegypti* and *Aedes albopictus*, which are widespread in tropical and subtropical regions [1]. Over the past two decades, recurrent outbreaks in Africa, Asia, Europe, and the Americas have underscored the epidemic potential of CHIKV and highlighted the urgent need for effective antiviral therapies and preventive strategies [1,2]. CHIKV infection is characterised by an acute febrile illness accompanied by severe polyarthralgia, myalgia, headache, fatigue, and skin rash. Although mortality rates remain relatively low, persistent joint pain and musculoskeletal complications may continue for months or even years after the acute phase of infection, significantly affecting the quality of life of affected individuals [3]. The socioeconomic burden associated with chronic chikungunya disease has become increasingly evident, particularly in endemic regions where healthcare resources are limited. Despite its growing global impact, no specific antiviral treatment has yet been approved for CHIKV infection, making a detailed understanding of its molecular biology essential for the development of targeted therapeutic interventions [4].

**Figure 1:**
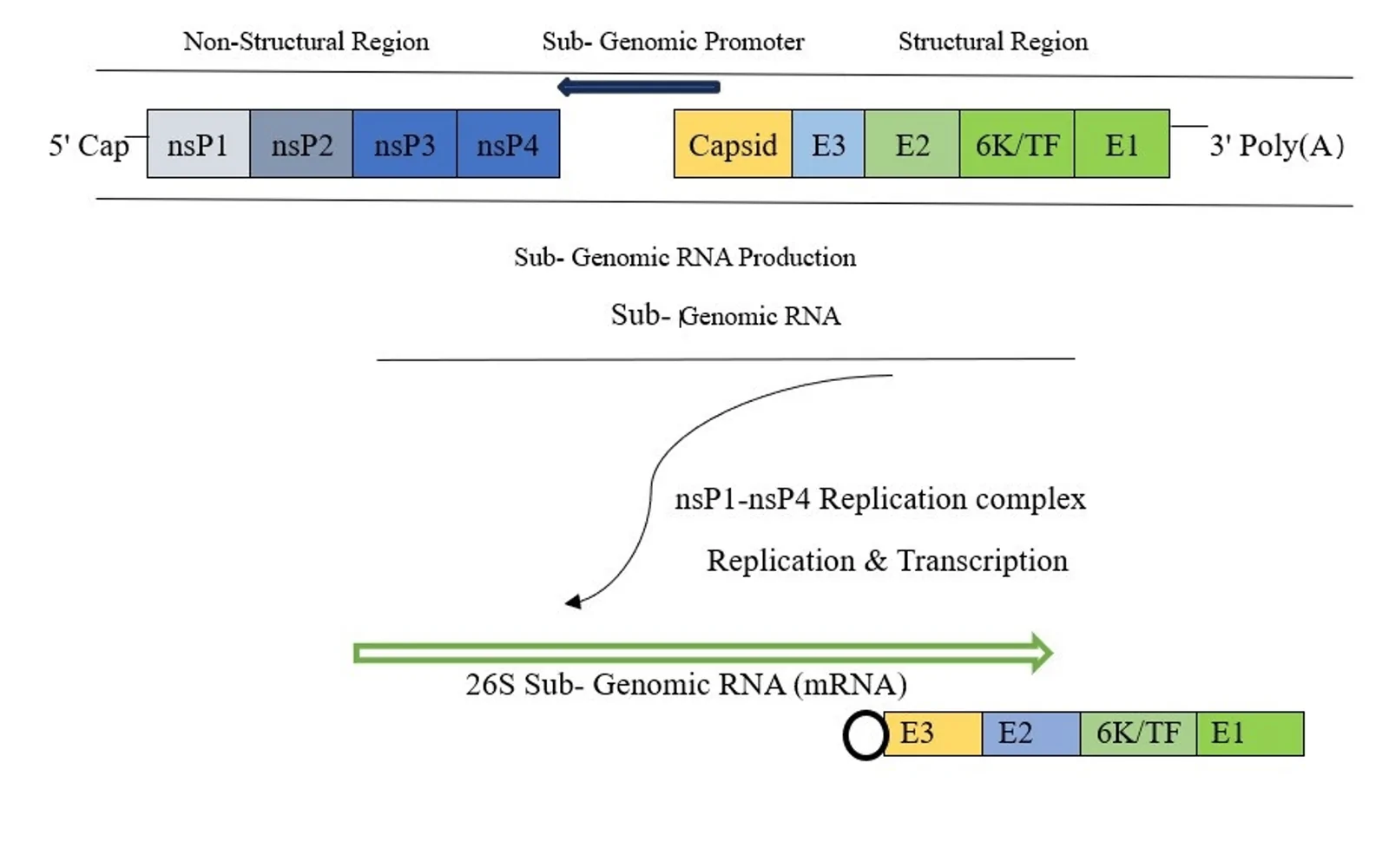
Genome organisation and expression of Chikungunya virus (CHIKV). Schematic representation of the CHIKV positive-sense single-stranded RNA genome showing the 5′ cap and 3′ poly(A) tail. The genome contains two major open reading frames (ORFs), where the 5′ ORF encodes the non-structural polyproteins (nsP1-nsP4), and the 3′ ORF encodes the structural polyprotein (C-E3-E2-6K/TF-E1). Following infection, the genomic RNA is translated to produce the replication machinery, which generates a negative-sense RNA intermediate that serves as a template for synthesis of new genomic RNA and subgenomic 26S RNA. Source: Figure created by the authors using Microsoft Word (Microsoft Corp., Redmond, WA, USA).

CHIKV possesses a positive-sense single-stranded RNA genome approximately 11.8 kb in length. The viral genome contains a 5′ methylated cap structure and a 3′ polyadenylated tail, allowing it to function directly as messenger RNA upon entry into the host cell [3]. Two major open reading frames encode the viral non-structural and structural proteins. The non-structural proteins (nsP1-nsP4) are responsible for RNA synthesis, genome replication, RNA capping, and modulation of host cellular pathways, whereas the structural proteins mediate viral assembly, entry, and release. Successful viral replication depends on the coordinated interaction of these proteins with host cellular factors and intracellular membranes [5]. Among the various stages of the CHIKV replication cycle, RNA capping, replication complex (RC) assembly, and translational regulation represent critical molecular events that determine viral fitness and pathogenicity. RNA capping, primarily mediated by the multifunctional enzyme nsP1, generates a type-0 cap structure at the 5′ end of viral RNAs [2]. This modification enhances RNA stability, promotes efficient translation, and enables the virus to evade host innate immune recognition. Simultaneously, viral replication occurs within specialised membrane-bound replication organelles known as spherules, where replication complexes composed of nsP1-nsP4 orchestrate the synthesis of genomic and sub-genomic RNAs [4]. Furthermore, CHIKV manipulates host translational machinery and antiviral signalling pathways to ensure preferential translation of viral proteins while suppressing host gene expression. Recent advances in structural biology, cryo-electron microscopy, and molecular virology have significantly improved our understanding of these interconnected processes. These discoveries have revealed several highly conserved viral enzymes and replication components as attractive antiviral targets [5]. This narrative review seeks to combine current knowledge about RNA capping, the assembly of the RC, and translational regulation. These interconnected molecular processes collectively control the replication of CHIKV, its ability to evade the immune system, and the synthesis of viral proteins. By understanding how these mechanisms interact, we can identify novel antiviral targets and guide the development of future therapeutic interventions against CHIKV. Figure 1 shows the genome organisation and expression of Chikungunya.

## Review

Search strategy

A narrative review was conducted to summarise current evidence on CHIKV RNA capping, RC assembly, and translational regulation. A narrative literature search was performed using PubMed, Scopus, Web of Science, and Google Scholar. The primary search period included studies published from January 2000 to March 2025, while older landmark publications were included to provide historical context on alphavirus molecular biology. The search strategy combined Medical Subject Headings (MeSH) terms and free-text keywords, including “Chikungunya virus,” “CHIKV,” “alphavirus,” “RNA capping,” “nsP1,” “methyltransferase,” “guanylyltransferase,” “replication complex,” “replication organelle,” “replication spherules,” “nsP2 protease,” “nsP3,” “stress granules,” “nsP4 polymerase,” “subgenomic RNA,” “26S RNA,” “translation,” “host shutoff,” and “innate immune response.” Boolean operators (AND/OR) were applied to refine database searches. Additional relevant studies were identified through manual screening of reference lists and citation tracking. Records were screened by title and abstract, and eligible full texts were assessed for relevance. Eligible studies included mechanistic studies, structural biology studies, in vitro studies, animal studies, antiviral screening studies, and relevant review articles that investigated CHIKV molecular biology, RNA capping, RC assembly, translational regulation, or antiviral development. Non-English articles, conference abstracts, unpublished reports, editorials without evidence, and purely clinical case reports were excluded. Duplicates were removed before full-text review. Data were extracted manually and synthesised narratively, and findings were organised thematically.

RNA capping and its biological significance in CHIKV

RNA capping is an essential post-transcriptional modification at the 5′ end of eukaryotic and some viral RNAs, involving the addition of a methylated guanosine through a 5′-5′ triphosphate linkage. This cap enhances RNA stability, facilitates translation, and helps viral RNAs avoid host immune detection. In positive-sense RNA viruses like CHIKV, capping is important as the viral genome directly functions as mRNA, efficiently recruiting host ribosomes for protein synthesis [6]. The canonical cap 0 structure (m⁷GpppN) is formed via enzymatic reactions: removal of the γ-phosphate by RNA triphosphatase, GMP transfer by guanylyl transferase, and methylation at the N7 position by methyltransferase. In some organisms, an additional 2′-O methylation creates a cap 1 structure (m⁷GpppNm), enhancing translation and evading innate immune sensors like RIG-I and MDA5 [7]. In CHIKV, the 5′ cap is a determinant of viral fitness. It promotes cap-dependent translation initiation by enabling recognition by the host eIF4E initiation factor, ensuring synthesis of viral non-structural and structural proteins [7]. The cap also protects viral RNA from degradation by host exonucleases, preserving genome integrity in the cytoplasm [8]. Mutations affecting the viral capping enzyme nsP1 impair cap formation, reduce translation efficiency, increase interferon induction, and attenuate viral replication [9,10].

Viral capping machinery and cap synthesis mechanism: nsP1 as the central enzyme

CHIKV RNA capping is primarily mediated by nsP1, which acts as the main enzyme responsible for generating the 5′ type-0 cap structure (m⁷GpppN) on both genomic and sub-genomic RNAs [7,8,11]. Unlike host mRNA capping, which involves multiple enzymes acting sequentially, CHIKV relies on nsP1 as a multifunctional capping factor that possesses both methyltransferase and guanylyltransferase activities [7,11]. In addition to its enzymatic roles, nsP1 associates with the plasma membrane and endosomal membranes, contributing to the formation of membrane-anchored replication spherules that provide a specialised environment for RNA synthesis and processing.

**Figure 2:**
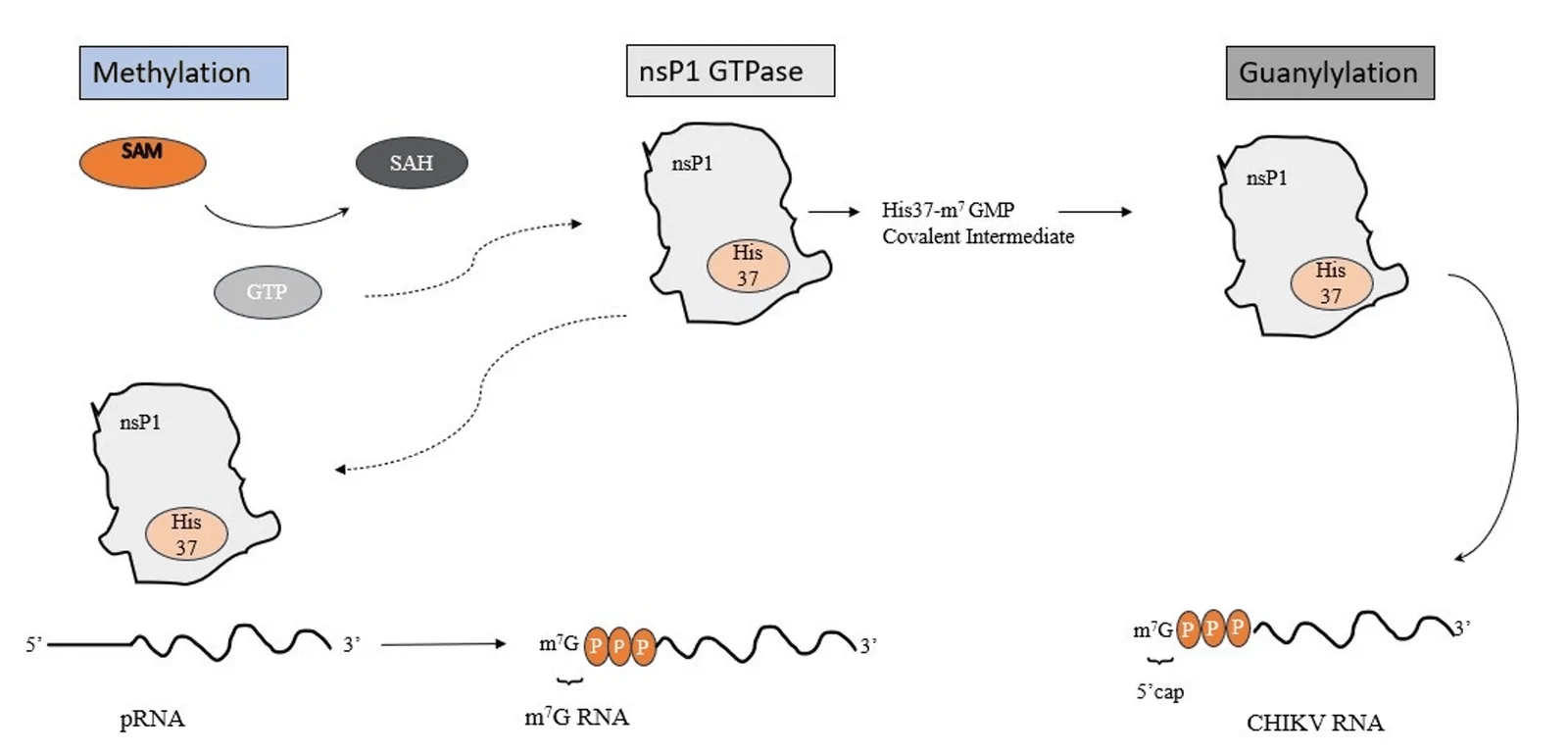
Chikungunya virus (CHIKV) RNA capping mechanism mediated by nsP1. Viral RNA is synthesised with a 5′ triphosphate end (pppN-RNA), which is converted into the capped Cap 0 structure (m⁷GpppN-RNA) through methyltransferase and guanylyltransferase activities using SAM as a methyl donor. RNA capping enhances RNA stability, supports efficient translation initiation, and contributes to evasion of host innate immune sensing mechanisms. SAM: S-adenosylmethionine; SAH: S-adenosylhomocysteine; GTP: guanosine triphosphate; nsP1: non-structural protein 1; His 37: histidine 37; m⁷GMP: 7-methylguanosine monophosphate, pRNA: precursor RNA Source: Original schematic created by the authors using Microsoft PowerPoint based on published mechanistic studies of alphavirus RNA capping. (Microsoft Corp., Redmond, WA, USA).

Alphavirus capping employs a unique “methylation-first” strategy in which nsP1 methylates GTP with S-adenosyl-L-methionine (SAM), producing 7-methylguanosine triphosphate (m⁷GTP) and S-adenosyl-L-homocysteine (SAH). The methylated nucleotide is then linked to a conserved histidine residue (His37) in the nsP1 active site, creating a transient m⁷GMP-enzyme intermediate [8,11]. Subsequently, nsP1 transfers the m⁷GMP moiety to the 5′ diphosphate end of the nascent viral RNA, generating the mature m⁷GpppN cap structure. This reversed order of reactions-methylation before guanylylation distinguishes alphaviruses from the canonical host capping pathway [12]. Figure 2 shows the CHIKV RNA capping mechanism mediated by nsP1 [13].

Membrane association

The membrane association of nsP1 is vital for organising CHIKV replication and capping machinery in specialised intracellular compartments. It anchors the RC to modified host membranes, such as those of the plasma membrane and endosomes, facilitating the formation of replication spherules [14,15]. These structures create a protected niche for viral RNA synthesis, shielding the viral genome from host defences [15]. The attachment of nsP1 to membranes is aided by an amphipathic α-helix at its C-terminus, which interacts with the lipid bilayer. Palmitoylation at conserved cysteine residues enhances nsP1's hydrophobic interactions, regulating its localisation and activity. If palmitoylation is lost or the helix is disrupted, it impairs membrane binding and reduces the efficiency of viral replication [14]. Membrane-bound nsP1 forms dodecameric oligomers that recruit other non-structural proteins and host factors necessary for RNA replication, ensuring efficient viral replication by coupling enzymatic function with spatial organisation [15].

Mechanism of cap synthesis

CHIKV RNA capping involves three reactions: (a) removal of the γ-phosphate by nsP2’s triphosphatase activity, (b) guanylylation mediated by nsP1 to form a 5′-5′ link, and (c) N7-methylation by nsP1 using SAM as a methyl donor [7,15]. This process converts the 5′ triphosphate end of viral RNA into a methylated cap structure (m⁷GpppN-RNA), important for RNA stability, translation, and immune evasion. It starts with nsP2 removing the γ-phosphate, creating a diphosphate intermediate (ppN-RNA). nsP1 then methylates GTP to form m⁷GTP and links it to produce an m⁷GMP-enzyme intermediate. nsP1 transfers the methylated guanine to the diphosphate RNA, yielding the m⁷GpppN cap [15]. This capping pathway allows for effective ribosome recruitment and helps evade immune detection by sensors like RIG-I and MDA5 [7].

Cap structure

The 5′ cap structure of CHIKV RNA acts as a molecular signature throughout the viral life cycle, facilitating efficient gene expression, RNA stability, and evasion of host immune defences. This methylated cap (m⁷GpppN-RNA) mimics host mRNAs, allowing CHIKV to utilise the host's translational machinery while avoiding activation of antiviral pathways [7]. The cap on viral RNA aids translation initiation by being recognised by eIF4E, which helps form the initiation complex. Without proper capping, viral RNAs have difficulty engaging with the host's translation machinery, leading to reduced protein production and viral replication. The cap protects CHIKV RNA from exonuclease degradation, preserving its integrity [15]. The N7-methylated guanine cap is important for immune evasion, as it hides viral RNA from cytoplasmic sensors, reducing interferon-mediated antiviral responses. Proper capping is essential for viral replication and pathogenicity; mutations in this process lower RNA stability and viral growth [11].

RC assembly and polyprotein cleavage switching in CHIKV

Replication of the CHIKV genome occurs within a highly organised membrane-associated RC, which serves as the central platform for viral RNA synthesis, sub-genomic transcription, and RNA processing [16,17]. This RC is primarily composed of the four viral non-structural proteins (nsP1-nsP4), together with host factors and remodelled intracellular membranes that provide a protected microenvironment for replication. Following viral entry and uncoating, the RNA genome functions directly as mRNA and is translated into polyprotein precursors (P123 and P1234) [18,19]. These polyproteins are subsequently processed by the viral nsP2 protease into mature nsP proteins, thereby enabling maturation of the functional RC. RC assembly occurs in invaginated membrane structures called spherules, derived mainly from the plasma membrane and endosomal compartments, which shield viral RNA replication intermediates from host innate immune sensors and enhance replication efficiency [17,19].

Each of the four CHIKV non-structural proteins (nsP1-nsP4) performs distinct but interconnected functions during viral replication. nsP1 mediates RNA capping and membrane anchoring, nsP2 possesses protease and helicase activities, nsP3 functions as a scaffold for host protein interactions, and nsP4 serves as the viral RNA-dependent RNA polymerase (RdRp). nsP1 acts as the membrane anchor and viral capping enzyme, promoting spherule formation through palmitoylation-dependent membrane association [12]. nsP2 possesses helicase and protease activities, enabling RNA unwinding and controlling polyprotein cleavage. nsP3 functions as a scaffolding protein that stabilises RC assembly and recruits host proteins such as G3BP to support replication. nsP4 serves as the RdRp and catalyses synthesis of both negative- and positive-sense viral RNA strands [19]. A regulatory checkpoint in CHIKV replication is the ordered cleavage of the non-structural polyproteins, which acts as a molecular switch controlling the transition from negative-strand to positive-strand RNA synthesis [18]. Early in infection, the RC predominantly contains uncleaved P123 associated with nsP4, forming an immature complex specialised for the synthesis of the complementary negative-strand RNA template [20]. As infection progresses, sequential cleavage of P123 by nsP2 generates mature nsP1-nsP4, producing a fully active RC that preferentially drives synthesis of positive-sense genomic RNA and transcription of the 26S subgenomic RNA required for structural protein expression [18]. 

The genome of CHIKV encodes four non-structural proteins (nsP1-nsP4) that play an important role in viral replication, as well as five structural proteins (Capsid, E3, E2, 6K/TF, and E1) that are responsible for virion assembly, maturation, and entry into host cells. A summary of the major functions of these proteins is provided in Table 1.

**Table 1:** Functional roles of Chikungunya virus (CHIKV) structural and non-structural proteins

Protein	Type	Function	Role in Viral Life Cycle
nsP1	Non-structural	RNA capping + membrane anchoring	Forms 5′ cap (m⁷GpppN), supports translation and immune evasion [21]
nsP2	Non-structural	Protease + helicase	Polyprotein cleavage, replication switching, host shutoff [22]
nsP3	Non-structural	Host interaction scaffold	Supports replication complex assembly, inhibits stress granules [23]
nsP4	Non-structural	RNA-dependent RNA polymerase (RdRp)	Negative-strand synthesis, genomic and 26S RNA production [21]
Capsid (C)	Structural	Nucleocapsid formation	Packages genomic RNA, forms viral core [24]
E3	Structural	Glycoprotein processing	Helps correct the folding of envelope proteins [23]
E2	Structural	Receptor binding	Responsible for host cell attachment [21,24]
6K	Structural	Membrane permeabilization	Supports glycoprotein trafficking and budding [22]
TF (Trans frame)	Structural	Virion release support	Enhances particle assembly and membrane curvature [23]
E1	Structural	Membrane fusion protein	Mediates fusion of the viral envelope with the host membrane [24]

RC localisation and RNA synthesis in CHIKV

Replication of CHIKV occurs within specialised membrane-bound RCs that form mainly on the plasma membrane and endosomal membranes [16]. These RCs concentrate viral non-structural proteins (nsP1-nsP4), host cofactors, and RNA intermediates, creating protected microenvironments that enhance replication efficiency while shielding viral RNA from host antiviral sensors, such as RIG-I and MDA5 [25].

**Figure 3:**
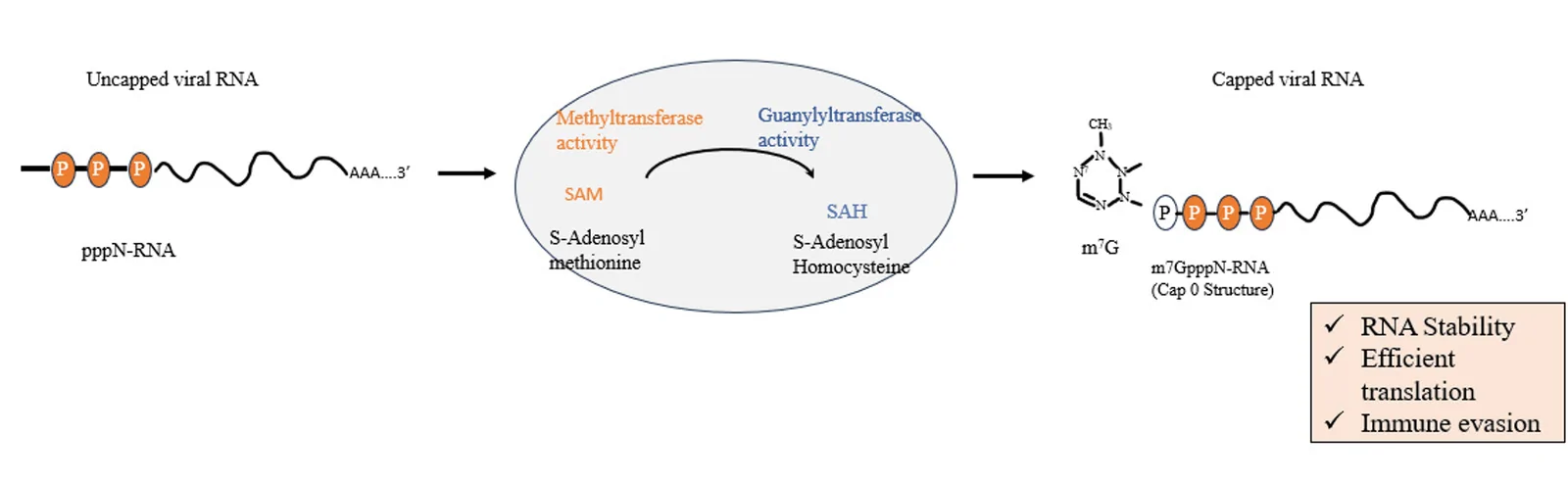
Assembly of the Chikungunya virus (CHIKV) replication complex on host membranes. Schematic representation of CHIKV replication complex formation on host intracellular membranes. Viral non-structural proteins nsP1-nsP4 associate with modified host membranes to form replication organelles (spherules), which serve as specialised compartments for viral RNA synthesis. Within these structures, negative-strand RNA intermediates are generated and used as templates for the production of genomic (+)RNA and subgenomic 26S RNA. Source: Figure created by the authors using Microsoft PowerPoint (Microsoft Corp., Redmond, WA, USA).

Spherule formation is initiated early in infection when nsP1 anchors the replication machinery to membranes through palmitoylation and electrostatic interactions, inducing membrane invagination to generate spherules (~50-70 nm) [16]. These vesicular structures function as replication factories, providing high local concentrations of enzymes and protecting replicating RNA from cytoplasmic nucleases [17]. During negative-strand synthesis, the early RC (P123-nsP4) produces a complementary negative-sense RNA template, which is essential for downstream RNA amplification. As infection progresses and polyprotein cleavage generates mature nsP proteins, the RC switches to positive-strand synthesis, producing new genomic RNA for packaging and translation [18]. Simultaneously, transcription of the 26S sub-genomic RNA is initiated from an internal promoter on the negative strand, ensuring high-level expression of structural proteins required for virion assembly [17]. The dynamic trafficking of RCs further regulates replication. Initially, replication occurs at the plasma membrane, but later RCs are internalised and accumulate in endosomal and lysosomal compartments, supporting coordination between RNA synthesis and virion assembly. Host lipid metabolism pathways (phosphatidylinositol and cholesterol) contribute to membrane curvature, while actin and microtubules assist in RC transport and spatial organisation [26]. CHIKV replication depends on compartmentalised RNA synthesis within spherules, enabling efficient genome amplification, immune evasion, and temporal regulation of viral gene expression [17]. Figure 3 shows the CHIKV RC on host membranes [23].

Host factors and replication fidelity in CHIKV

CHIKV replication relies on host cellular factors for RC assembly, membrane remodelling, and RNA synthesis. Essential host membrane lipids like phosphatidylserine and cholesterol support nsP1-mediated membrane anchoring and spherule formation, while cytoskeletal components, such as actin filaments and microtubules, aid in intracellular trafficking and organisation of replication sites [6,14]. Molecular chaperones like Hsp70 and Hsp90 aid in folding viral non-structural proteins, while RNA-binding proteins like G3BP1/2 interact with nsP3 to enhance replication and modulate stress granules. Host enzymes like SAM synthetase supply substrates for nsP1-dependent RNA capping [27,28]. Replication fidelity is primarily determined by nsP4, the viral RdRp, which lacks proofreading activity and therefore generates mutations at an estimated rate of 10⁻⁴-10⁻⁵ substitutions per nucleotide per cycle [29]. This controlled error rate supports viral adaptability while maintaining sufficient genome stability for productive infection [30].

Translation of genomic RNA in CHIKV

Upon entry into a host cell, the CHIKV genomic RNA acts as messenger RNA for synthesising viral non-structural proteins necessary for replication. Translation is mainly cap-dependent, utilising host ribosomes and initiation factors. The viral genome features a type-0 5′ cap (m⁷GpppN) and a 3′ poly(A) tail, similar to cellular mRNA, which aids in recruiting the host's translational machinery [31]. The 5′ cap is recognised by the eIF4F complex, where eIF4E binds the cap, eIF4G acts as a scaffold linking the RNA to the 40S ribosomal subunit through eIF3, and eIF4A functions as a helicase to unwind RNA secondary structures and support ribosomal scanning [32].

The 43S pre-initiation complex scans the 5′ untranslated region until the first AUG start codon, located at the beginning of the nsP1 coding region, is recognised within a favourable Kozak context [33]. CHIKV synthesises the viral non-structural polyprotein as either P123 or the longer P1234 by occasionally reading through an opal stop codon at the end of nsP3 to include nsP4. This process regulates nsP4 abundance, ensuring proper RC stoichiometry [31]. The polyproteins are anchored to membranes via nsP1 and cleaved by the nsP2 protease into mature nsP1-nsP4, linking translation directly to RC assembly and initiating viral RNA synthesis [34].

Translation of 26S sub-genomic RNA

In the late stage of CHIKV infection, the 26S sub-genomic RNA, transcribed from an internal promoter on the negative-strand RNA, drives the synthesis of structural proteins. This sub-genomic RNA acts as an independent mRNA with a 5′ cap and a 3′ poly(A) tail, facilitating efficient translation by host ribosomes. High levels of 26S RNA promote the production of structural proteins needed for virion assembly [35]. Translation of the 26S RNA produces a single structural polyprotein arranged as Capsid-pE2-6K-E1. The capsid protein is rapidly released by autocatalytic cleavage and forms the nucleocapsid core by packaging genomic RNA. The remaining polyprotein is processed within the endoplasmic reticulum and Golgi apparatus by host signal peptidases and furin-like proteases, generating mature envelope proteins E2 and E1 [36]. These glycoproteins form heterodimers that are essential for receptor binding, membrane fusion, and virion budding. The small 6K protein supports glycoprotein folding and trafficking, while a programmed frameshift can produce the TF protein, which enhances membrane curvature and viral release [24]. Translation of 26S RNA is tightly regulated and becomes dominant as the virus shifts from genome replication to particle assembly, ensuring efficient production of structural components even during host translational shutoff [37].

Host translation shutoff and immune evasion strategies in CHIKV

CHIKV ensures efficient viral protein production by inducing host translation shutoff, a strategy that suppresses cellular gene expression while maintaining active translation of viral RNAs. This selective control redirects ribosomes and translation initiation factors toward viral genomic and sub-genomic RNAs, enabling high-level synthesis of proteins required for replication and virion assembly [38]. A contributor to host shutoff is nsP2, which translocates into the nucleus and disrupts host transcription by targeting RNA polymerase II, thereby reducing the availability of host mRNAs for translation. This transcriptional suppression also limits interferon-stimulated gene expression, weakening antiviral signalling and creating a favourable environment for viral replication [39].

**Figure 4:**
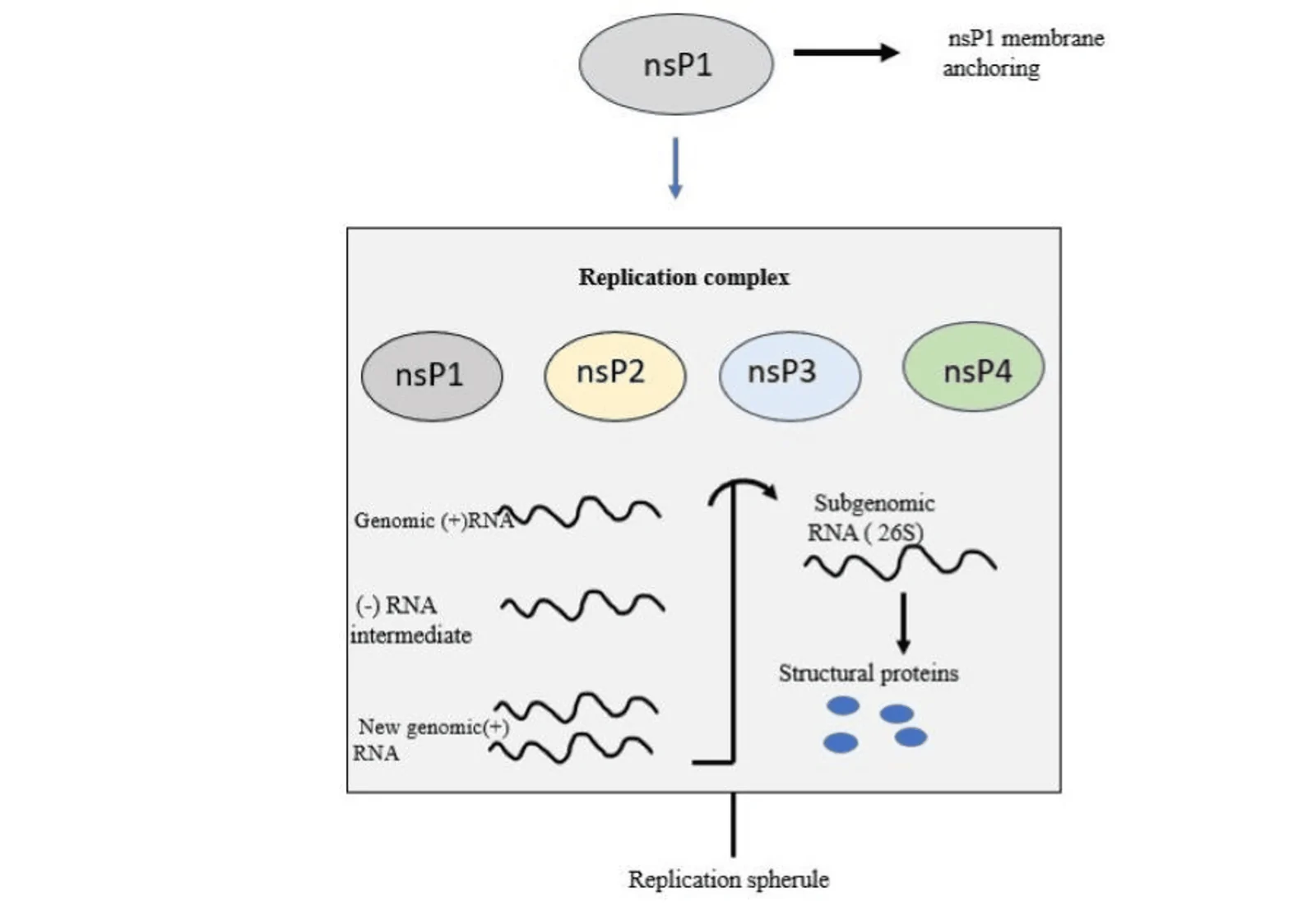
Translational regulation and host shutoff mechanisms during Chikungunya virus (CHIKV) infection. Viral capped RNAs efficiently recruit host translation initiation factors (eIF4E, eIF4G, eIF4A) to support viral protein synthesis. In parallel, CHIKV suppresses host gene expression through mechanisms including nsP2-mediated transcriptional shutoff and phosphorylation of eIF2α, thereby reducing host protein synthesis while promoting viral replication and immune evasion. nsP: non-structural protein Source: Figure created by the authors using Microsoft PowerPoint (Microsoft Corp., Redmond, WA, USA).

CHIKV genomic and 26S sub-genomic RNAs possess a 5′ cap and poly(A) tail, allowing them to efficiently recruit the eIF4F translation initiation complex and directly compete with host transcripts. The high abundance of viral RNAs further enhances their dominance in ribosome recruitment. CHIKV also counteracts cellular stress responses that normally inhibit translation. The viral protein nsP3 binds G3BP1/2, preventing the formation of stress granules that would otherwise sequester translation factors and suppress viral mRNA translation [39]. CHIKV can overcome antiviral translation blocks triggered by dsRNA-sensing pathways, including PKR-mediated phosphorylation of eIF2α, ensuring continued viral protein synthesis even under strong host stress conditions. CHIKV uses nsP1-mediated RNA capping to mimic host mRNA and avoid restriction by IFIT proteins, which preferentially inhibit translation of uncapped or improperly capped RNA. Together, these mechanisms allow CHIKV to sustain viral translation, suppress host antiviral responses, and efficiently coordinate the shift from early non-structural protein synthesis to late-stage structural protein production [39]. Translational regulation and host shutoff mechanisms during CHIKV infection are shown in Figure 4 [38].

Integration of RNA capping, replication, and translation in CHIKV

The CHIKV life cycle depends on the tight coordination of RNA capping, genome replication, and translation, ensuring efficient viral gene expression while limiting activation of host antiviral responses. These processes are functionally linked through the activities of the non-structural proteins (nsP1-nsP4), which regulate both RNA synthesis and its maturation into translation-competent molecules [13].

During infection, translation of the incoming genomic RNA occurs first, producing the non-structural polyprotein required to assemble RCs. Once sufficient nsPs accumulate, replication becomes dominant, with early RCs synthesising negative-strand RNA templates followed by large-scale production of positive-sense genomic and subgenomic RNAs. Newly synthesised RNAs are rapidly capped by nsP1, generating an m⁷GpppN structure that stabilises the RNA and promotes efficient recruitment of host translation initiation factors. Co-transcriptional capping also prevents accumulation of uncapped RNA, thereby reducing recognition by innate immune sensors such as RIG-I and IFIT proteins [6]. Spatial compartmentalisation further supports this integration. Replication and capping occur inside membrane-derived spherules, which protect viral RNA intermediates and concentrate enzymes for efficient synthesis. Mature capped RNAs are then released into the cytoplasm for translation or packaged into progeny virions. CHIKV shifts from early non-structural protein synthesis to mid-phase RNA amplification and finally to late-stage translation of 26S subgenomic RNA for structural protein production, ensuring successful virion assembly and release [15].

Current challenges in targeting CHIKV replication machinery

Despite significant advances in understanding CHIKV molecular biology, several challenges continue to limit the development of effective antiviral therapies. The high mutation rate associated with the error-prone nsP4 RdRp contributes to viral adaptability and may facilitate the emergence of antiviral resistance. Many viral non-structural proteins interact extensively with host pathways, making selective inhibition difficult without causing host toxicity. Structural flexibility within RCs and limited availability of high-resolution dynamic models further complicate rational drug design. Although several experimental compounds targeting nsP1 capping activity and nsP2 protease function have demonstrated promising in vitro antiviral activity, few candidates have advanced to clinical evaluation. The absence of universally accepted animal models and the limited understanding of chronic chikungunya pathogenesis remain important barriers for translational research. Addressing these limitations through integrated structural, computational, and functional studies will advance the development of CHIKV antivirals.

Future perspectives and antiviral opportunities

CHIKV continues to pose a major public health challenge due to the absence of specific antiviral therapies and the expanding geographical distribution of competent mosquito vectors. Recent advances in cryo-electron microscopy and structural virology have significantly improved understanding of the alphavirus replication machinery, particularly the architecture of nsP1 capping pores and replication spherules. These findings provide opportunities for structure-guided antiviral drug development targeting conserved non-structural proteins. Among the viral proteins, nsP1 has emerged as a promising antiviral target because of its essential role in RNA capping, membrane association, and RC assembly [40]. Small-molecule inhibitors targeting nsP1 methyltransferase and guanylyltransferase activities have shown encouraging antiviral effects in experimental studies. Similarly, nsP2 protease and helicase domains represent attractive targets for inhibitors aimed at disrupting polyprotein processing and host shutoff mechanisms. The RdRp nsP4 also remains an important therapeutic target, although its structural flexibility and high mutation rate continue to complicate inhibitor development [41].

Host-directed antiviral approaches are also being explored, including modulation of lipid metabolism, stress granule pathways, and host translation factors required for efficient viral replication. Although these strategies may reduce the likelihood of antiviral resistance and provide broader antiviral activity, they may also interfere with essential host cellular pathways, potentially resulting in off-target effects or toxicity. Therefore, careful target selection and comprehensive preclinical safety evaluation are essential before clinical application [40]. Advances in mRNA vaccine technology, nanoparticle-based delivery systems, and broad-spectrum antiviral compounds may contribute to future chikungunya prevention and treatment strategies. Continued integration of structural biology, molecular virology, and antiviral screening studies will be essential for developing effective therapeutic interventions against CHIKV and related alphaviruses [42].

Limitations

This review is based on published English-language literature retrieved from major scientific databases and may not include unpublished studies, regional reports, or non-indexed literature. As rapidly evolving structural and antiviral studies on CHIKV continue to emerge, some recently developed findings may not have been fully represented at the time of article preparation. In addition, variations in experimental models, viral strains, and methodological approaches among published studies may influence the interpretation and comparability of reported findings. Despite these limitations, the review provides an integrated overview of current knowledge regarding CHIKV RNA capping, replication, and translational regulation.

## Conclusions

CHIKV depends on tightly coordinated RNA capping, genome replication, and translation to ensure efficient infection and viral persistence. The multifunctional nsP1 is central to this process, mediating methylation-first RNA capping and anchoring replication complexes to host membranes to form protected replication spherules. Regulated polyprotein cleavage by nsP2 controls the switch from early negative-strand synthesis to late-stage amplification of genomic and 26S subgenomic RNAs, while nsP4 functions as the RdRp driving RNA synthesis and contributing to viral evolution. CHIKV also modulates host translation and innate immune pathways through host shutoff and stress granule inhibition to favour viral protein production. Recent structural and cryo-electron microscopy studies have provided key insights into replication factory architecture, supporting structure-guided development of antivirals targeting nsP1, nsP2, and nsP4.
